# Ex Vivo Transdermal Delivery of Nicotinamide Mononucleotide Using Polyvinyl Alcohol Microneedles

**DOI:** 10.3390/polym15092031

**Published:** 2023-04-25

**Authors:** Farzaneh Sabbagh, Beom-Soo Kim

**Affiliations:** Department of Chemical Engineering, Chungbuk National University, Cheongju 28644, Republic of Korea; f.sabbagh@chungbuk.ac.kr

**Keywords:** nicotinamide mononucleotide, transdermal delivery, patch, polyvinyl alcohol, carboxymethyl cellulose

## Abstract

Nicotinamide mononucleotide (NMN), which has recently been spotlighted as an anti-aging agent, is a precursor of the coenzyme nicotinamide adenine dinucleotide that plays an important role in intracellular redox reactions. NMN capsules for oral administration currently on the market have a problem in that they are almost fully metabolized in the stomach and liver and excreted as nicotinamide. Therefore, there is a need to develop a patient-friendly delivery method that can improve the bioavailability of NMN. For this purpose, various polyvinyl alcohol (PVA)-based microneedle patches were fabricated to develop a transdermal delivery system for NMN. First, the molecular weight effect of PVA on the shape and microstructure of microneedles was studied. After selecting the optimal molecular weight PVA, the swelling of the microneedles and the ex vivo release of NMN were studied. The effect of carboxymethyl cellulose (CMC) and dimethyl sulfoxide on NMN release was also investigated. The highest NMN release of 91.94% in 18 h was obtained using a 9.5 kDa molecular weight PVA microneedle containing NMN and CMC.

## 1. Introduction

Microneedle systems are becoming increasingly popular due to technological advances in drug delivery applications and can offer many advantages over other delivery routes. Advantages of transdermal delivery using microneedles include improved patient compliance, no interaction with gastrointestinal fluids, prevention of first-pass metabolism, and painless self-administration [1,2]. Unlike reusable stainless steel hypodermic needles, the use of dissolvable polymer microneedles eliminates the creation of hazardous sharp waste, preventing the potential for injury and transmission of bloodborne infections such as hepatitis and human immunodeficiency virus [3]. To date, numerous materials have been tested on microneedles, including polymers and composites. The manufacture of microneedles requires the use of materials that can come into contact with human tissue without infection or tissue damage. Therefore, significant advances have been made in materials for microneedles such as silicon, metals, glass, ceramics, etc. Several natural or synthetic polymers have been developed to produce polymeric microneedles such as polyhydroxyalkanoate [4], hyaluronic acid [5], chitosan [6], polyglycolic acid [7], chitin [8], polylactic acid [9], silk fibroin [10], and polyvinyl alcohol (PVA) [11]. Among these, the use of PVA is of increasing interest in medical applications such as drug delivery, dressing, soft biomaterial implantation, and targeted tissue transport systems [11].

Nicotinamide mononucleotide (NMN), which has recently been spotlighted as an anti-aging agent, is a precursor of the coenzyme nicotinamide adenine dinucleotide (NAD^+^) that plays an important role in intracellular redox reactions [12]. NMN is made by the reaction of a phosphate group with a nucleoside composed of nicotinamide (NAM) and ribose [13]. NMN has two anomeric forms, including α and β, with β-NMN being the active form. Various types of whole foods, such as fruits, vegetables, and meat, contain small amounts of NMN [14]. As we age, our bodies produce less NAD^+^ and communication between mitochondria and cell nuclei is impaired, and a decrease in NAD^+^ reduces the cells’ ability to produce energy, which can lead to aging and disease [14,15]. When NMN enters the body, it is converted to NAD^+^ and the amount of NAD^+^ in the body increases, suggesting that aging can be minimized. Therefore, NMN can be commercialized in various fields such as the treatment for each disease, nutritional food related to longevity, and cosmetics for anti-aging. The NAD^+^ molecule itself is too large to be fully absorbed through the digestive system when taken in capsules. Although NMN and nicotinamide riboside (NR) are used as supplements to increase NAD^+^ levels in the body, NMN capsules for oral administration currently on the market are partially digested in the stomach and are almost fully metabolized in the liver and excreted as NAM [16]. Intravenous infusion of NAD^+^ has been reported to increase NAD^+^ levels in human plasma by 400% [17]. Therefore, there is a need to develop a patient-friendly delivery method that can improve the bioavailability of low molecular weight, hydrophilic NMN molecules instead of intravenous injection. 

The skin protects the body from infection and foreign substances [18]. For drug delivery via the skin route, transdermal delivery is the best way to maintain an effective concentration at a specific time and intended rate. By avoiding first-pass metabolism and increasing patient compliance, transdermal delivery may take precedence over oral and injection routes [19,20]. When microneedles loaded with NMN are injected into the skin, NMN can be released from the dermis and distributed throughout the body through capillaries and blood vessels [21]. 

In this study, we fabricated PVA microneedles by loading NMN into the microneedle tip. To study the transdermal delivery of NMN, the ex vivo release of NMN from microneedles was investigated using the Franz cell diffusion system. The swelling and ex vivo release of NMN were investigated by changing the microneedle composition. The skin penetration and drug delivery ability of the manufactured microneedles was verified through the microneedle injection into porcine ear skin, which has biochemical similarities to human skin. This is the first report on a transdermal NMN delivery system using microneedles.

## 2. Materials and Methods

### 2.1. Chemicals

Polyvinyl alcohol (PVA) with different average molecular weights (9.5, 18, 50, 93.5, 166 kDa) and sodium carboxymethyl cellulose (CMC) were purchased from Sigma-Aldrich., Saint Louis, MO, USA. β-Nicotinamide mononucleotide (NMN) was purchased from Tokyo Chemical Industries Co., Tokyo, Japan. Nicotinamide (NAM) and dimethyl sulfoxide (DMSO) were purchased from Samchun Co., Seoul, Republic of Korea. Silicone templates, spring applicators, casings, and casing inserts were purchased from Micropoint Technologies PTE LTD, Singapore, Singapore. Distilled water was used in all synthesis and analysis procedures. All chemicals were used as received.

### 2.2. Preparation of Microneedles

In the first step, three different microneedle patches were fabricated for different drug concentrations. Initially, PVA solutions (20 g/100 mL water) were prepared and then mixed with different amounts of NMN (2.9, 5.8, 8.8% of PVA). The mixture of PVA and NMN was placed on a magnetic stirrer for 2 h at 50 °C to obtain a homogeneous solution. For each fabrication, 200 μL of the NMN-polymer mixture was applied to a silicone mold and maintained in an oven at 50 °C for 2 h to form a microneedle tip. After drying the drug loaded layer, 100 μL PVA solution without NMN was applied to the mold to form the outer layer. After peeling the mold, it was dried in an oven and stored overnight in a desiccator at room temperature until the experiment was carried out [22].

Multilayer microneedles were fabricated in the same manner using different PVA molecular weights (9.5, 18, 50 kDa). Four microneedles with different compositions (NMN-PVA, NMN-PVA-CMC, NMN-PVA-DMSO, NMN-PVA-CMC-DMSO) were also fabricated. In this fabrication, NMN, CMC, and DMSO were used as 2.9%, 1.8%, and 128% of PVA, respectively. In addition, microneedle patches were prepared by changing the CMC concentration (0%, 2.9% of PVA).

The surface morphology of the microneedle patch was analyzed using a field emission scanning electron microscope model ULTRA PLUS (LEO-1530, Zeiss, Oberkochen, Germany). The resolution was 0.8 nm at 15 kV and 1.6 nm at 1 kV, vacuum 10^−5^ Torr. Elastically scattered electrons were detected using a backscattered electron detector. Samples were coated with platinum in a sputtering device with an accelerator voltage of 3 kV.

### 2.3. Patch Thickness

The thickness of the microneedle patch was measured with a Mitutoyo digital micrometer (model 293-821-30, Tokyo, Japan) with an accuracy of ±2 μm. The experiment was performed three times [23].

### 2.4. Swelling of Microneedle Patches

The microneedle patch was immersed in pseudo-extracellular fluid (PECF) buffer at pH 8.5 and 37 °C to swell to reach equilibrium. The change in swelling ratio (SR %) with time was determined by evaluating the weight of the sample at time increments and calculated using Equation (1).

(1) $$\mathrm{SR}\;\%=[\frac{\mathrm{Weight}\;\mathrm{of}\;\mathrm{the}\;\mathrm{sample}\;\mathrm{at}\;\mathrm{time}\;t\;-\mathrm{Weight}\;\mathrm{of}\;\mathrm{the}\;\mathrm{sample}\;\mathrm{at}\;\mathrm{time}\;0\;}{\mathrm{Weight}\;\mathrm{of}\;\mathrm{the}\;\mathrm{sample}\;\mathrm{at}\;\mathrm{time}\;0}]\;\times 100$$

Before measuring the weight of the sample, surface water was removed using filter paper [24]. The average of three readings was reported for each experiment.

### 2.5. Determination of NMN Content in Microneedle

To determine the content of NMN loaded into the microneedle, the tip of a representative patch was placed in 1.0 mL of PECF buffer solution, followed by the vortex mixing to completely release the encapsulated drug and filtered through a 0.45 μm microporous membrane filter [25]. After centrifugation at 7168× *g* for 10 min, the supernatant was separated and analyzed by HPLC. This experiment was performed in triplicate. The percentage of entrapped NMN was calculated as:

(2) $$\mathrm{Entrapment}\;\mathrm{Efficiency}\;(\%)=\;\frac{\mathrm{Amount}\;\mathrm{of}\;\mathrm{drug}\;\mathrm{entrapped}}{\;\mathrm{Total}\;\mathrm{amount}\;\mathrm{of}\;\mathrm{drug}\;\mathrm{taken}}\times 100$$

### 2.6. Ex Vivo Release Studies

PECF buffer mimicking interstitial fluid (pH~8) was prepared by adding 0.25 g KCl, 0.68 g NaCl, 0.32 g NaH_2_PO_4_, and 2.5 g NaHCO_3_ to 100 mL of distilled water [26]. Then, the solution was stirred until all salts were completely dissolved and the solution was filtered through a 0.22 µm filter to remove particulate matter. Quantification of NMN release from microneedle patches was evaluated by incubating NMN-loaded microneedles in an 8 mL Franz cell diffusion device containing PECF medium and porcine ear skin as the biological membrane at 37 ± 0.5 °C and 50 rpm. A 500 μL PECF sample containing released NMN was collected and replaced with 500 μL fresh medium. Sampling was done through the side arm of the cell using a long syringe at specific time intervals. All samples were analyzed by high pressure liquid chromatography (HPLC, YL 9100, Younglin Inc., Anyang, Republic of Korea). All solutions were filtered using a 0.22 μm membrane. NMN concentration was quantified using an Optima Pak C18 column (250 × 10 mm, RS Tech, Daejeon, Republic of Korea). Isocratic elution was performed using a mobile phase consisting of acetonitrile (10%, *v*/*v*) and water (90%, *v*/*v*) at a flow rate of 1.0 mL/min. The NMN peak was detected at 260 nm using a UV-Vis detector [27]. NMN was quantified from the calibration curve y = 6354.6x − 401.43 (R^2^ = 0.99), where x is the concentration of NMN in mg/mL (0 to 3.34) and y is the peak area. NAM was also quantified using HPLC from the calibration curve y = 6426.7x + 113.82 (R^2^ = 0.99), where x is the concentration of NAM in mg/mL (0 to 1.12) and y is the peak area. All cumulative NAM releases were expressed in mg (not %) because NAM was produced from NMN.

## 3. Results and Discussion

### 3.1. Preparation and Injection of Microneedles

**Figure 1:**
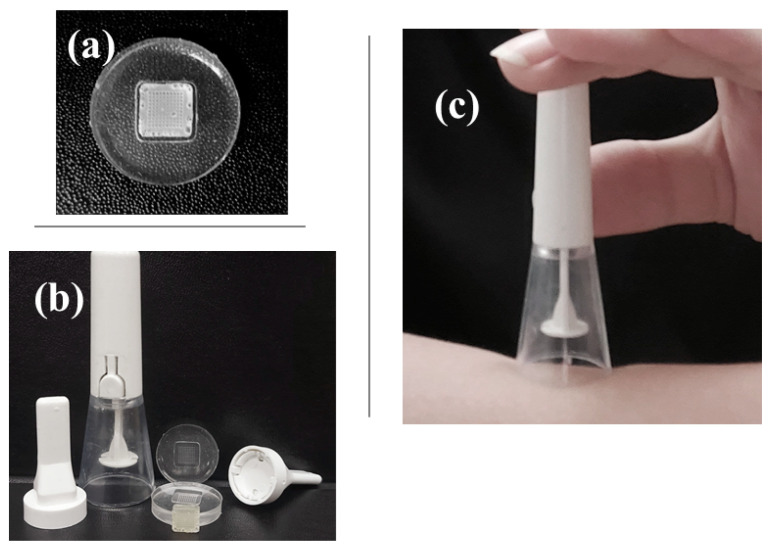
Photographs of (**a**) a silicone mold containing a microneedle loaded with NMN and (**b**) an applicator used to apply the microneedle patch to the skin; (**c**) The microneedle patch was first attached to the applicator, and then activated by pressing the white button to release the microneedle.

An image of the silicone mold is shown in Figure 1a. The silicone mold is composed of a patch size of 8 mm × 8 mm, a height of 500 μm, a spacing of 500 μm, a base of 200 μm × 200 μm, and an array of 10 × 10. To make the microneedle tip, a polymer composite material was fabricated and injected into a mold. As shown in Figure 1b, the microneedle patch was injected into the porcine skin using an applicator. A casing insert shown in Figure 1b was used to install the microneedle patch on the applicator without damaging the microneedle patch [18]. Ex vivo microneedle insertion was performed by piercing an NMN-loaded microneedle into excised porcine skin, an ideal skin model for microneedle insertion. With the aid of an applicator specifically designed for microneedle insertion (Figure 1c), the microneedle patch easily penetrated the porcine skin. To ensure that the microneedle was fully inserted into the skin, the applicator was activated by pressing the trigger button and holding the device in place for at least 10 s [28]. This holding time is important for the microneedles to dissolve into the skin. Upon contact with interstitial fluid (the fluid that fills the spaces between cells), the microneedle tip rapidly dissolves and releases drugs [29]. This microneedle system can penetrate the skin epidermis and deliver drugs contained in a polymer matrix. McGrath et al. [30] used an applicator to investigate the effect of the soluble microneedle composition on skin penetration.

### 3.2. Scanning Electron Microscopy Studies

**Figure 2:**
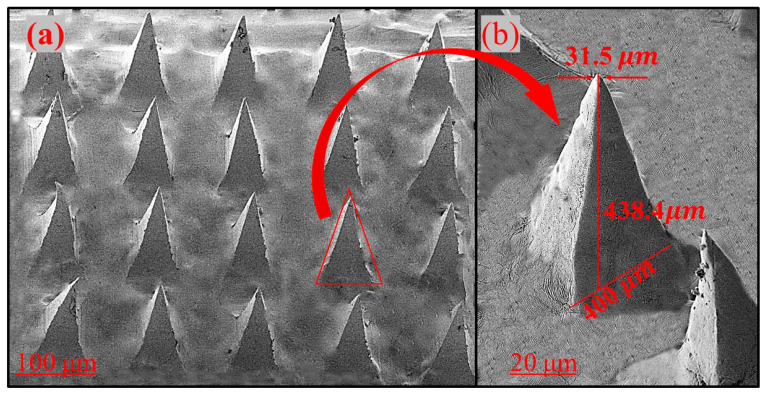
Scanning electron microscopy images of (**a**) PVA microneedles containing NMN, and (**b**) single PVA microneedle from the PVA microneedle array (molecular weight of PVA: 9.5 kDa).

An SEM image of the microneedle is shown in Figure 2. Dissolvable microneedles have a sharp tip and well-defined edges. Conical needles are uniformly and regularly arranged on the surface (Figure 2a). The average tip diameter, base diameter, and height of the microneedles were 31.5, 400, and 438 µm, respectively. For transdermal delivery through the stratum corneum, this height is suitable for microneedle penetration. SEM results from other studies are also consistent with these values [31], suggesting that the tip is suitable for injection into the skin. In a study conducted by Zhao et al. [32], tetramethyl pyrazine-loaded dissolving microneedle patches were fabricated. The microneedles had a conical shape, sharp tips, and a smooth surface. It was also found that the height of the needle was about 800 μm and the diameter of the base was about 300 μm. In another study by Khalid et al. [33], a microneedle patch of thiolated chitosan (TCS) and polyvinyl acetate was fabricated for the systemic delivery of dydrogesterone. TCS-polyvinyl acetate-based microneedle patch had a length of 575 μm with a sharp pointed end.

**Figure 3:**
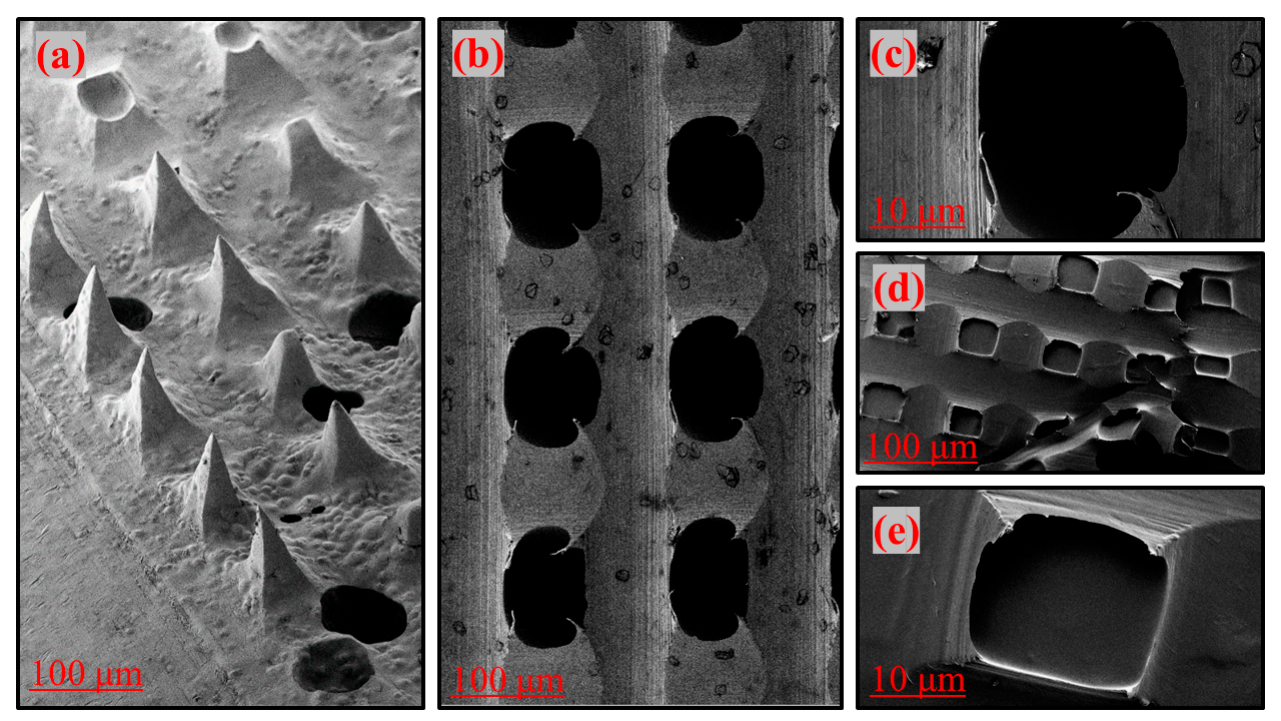
Scanning electron microscopy images of PVA microneedles containing NMN. (**a**–**e**) shows inappropriate microneedle tips due to high molecular weight PVA (molecular weight of PVA: 93.5 (**a**,**b**) and 166 kDa (**c**–**e**).

The properties of microneedles depend on the molecular weight of PVA [34]. PVA crystallites act as physical crosslinking points in the polymer matrix [35]. Increasing the molecular weight or viscosity of PVA in the patch formulation increases the viscosity of the gel layer, which slows drug dissolution [36]. Drug release depends on PVA matrices with different molecular weights, enabling the structure of different patches with controlled and sustained release [37]. Low molecular weight PVA (Figure 2) helps with sharper microneedle tips, higher swelling, and faster drug release [38]. High molecular weight polymers have problems with high diffusivity in the skin [39]. As shown in Figure 3a to Figure 3e, the high molecular weight PVA was unable to fill the cavity of the mold to make the tip, and the patch was full of pores (Figure 3a–c) and incomplete tips (Figure 3d,e). As a result, the low molecular weight PVA (9.5, 18, 50 kDa) could be easily injected into the skin using a microneedle. High molecular weight PVA (93.5, 166 kDa) is available as a gel that can be placed on body sites and solidified. PVA with a molecular weight of 166 kDa was not able to form needles and was therefore not used in this study.

**Figure 4:**
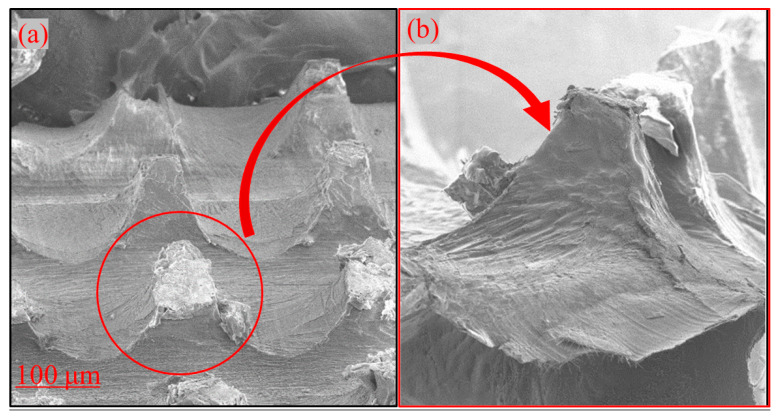
Scanning electron microscopy images of dissolving microneedles. (**a**) Microneedles in a 10 × 10 microneedle array after injection, (**b**) individual microneedle tip after injection into porcine ear skin using an applicator.

Microneedles are used to create microscopic channels in the skin using the poke and patch method through which drugs can easily transfer from the polymer patch into the skin [39,40]. The advantage of this method is to increase drug permeability [39,40]. In this regard, a PVA microneedle with an array of ~440 μm in length was pressed vertically for approximately 10 s using an applicator and injected through the stratum corneum of the skin, and a channel was created to deliver the NMN-loaded microneedles to the skin. Figure 4a shows the microneedle tip after injection into the skin. The microneedle can penetrate the skin, but as shown in Figure 4b, the tip is broken due to the flexibility of the skin.

### 3.3. Entrapment Efficiency of NMN in Microneedle

The NMN-PVA-CMC microneedle was used as a representative sample to perform the experiment. The relative content of each microneedle normalized to the labeled amount (5 mg) was 99.83 ± 0.06%. The hydrophilic nature of the entrapped NMN is responsible for the high % entrapment efficiency of the optimized formulation. The physicochemical properties of the drug, such as solubility and molecular weight, can affect its ability to be entrapped within microneedles [41].

### 3.4. Ex Vivo Release Studies

When PVA, a hydrophilic polymer, is placed in an aqueous liquid, it absorbs the liquid medium and hydrates to form a gel layer [42]. Two important and effective factors for drug release from PVA are gel diffusion barrier and surface erosion [41]. Leaching of the PVA surface can lead to the initial burst of NMN as a hydrophilic drug. Glassy polymer matrices containing swelling agents rapidly change from a glassy to a rubbery state, which is associated with swelling [42,43]. The polymer chains in PVA initially absorb water undisturbed, but as they become more solvated, they expand the distance and amplitude between each other. A decrease in the polymer transition temperature is associated with a decrease in the concentration of the swelling agent, which is influenced by the temperature and thermodynamic interactions of the polymer-water system [43]. There is a clear contrast between the rubbery and glassy regions, and the matrix swells with the increasing volume. The gel layer gradually thickens as water penetrates deeper into the matrix. Meanwhile, the outer layer is completely hydrated and begins to dissolve or erode. As the water reaches the center of the system, the drug concentration decreases below the solubility value and the rate of drug release begins to decrease. As the outer layer thickens over time, the diffusion path length decreases. The porous core of the inert matrix is eroded or dissolved by the elution medium. This porous network carries soluble drugs to the surface.

**Figure 5:**
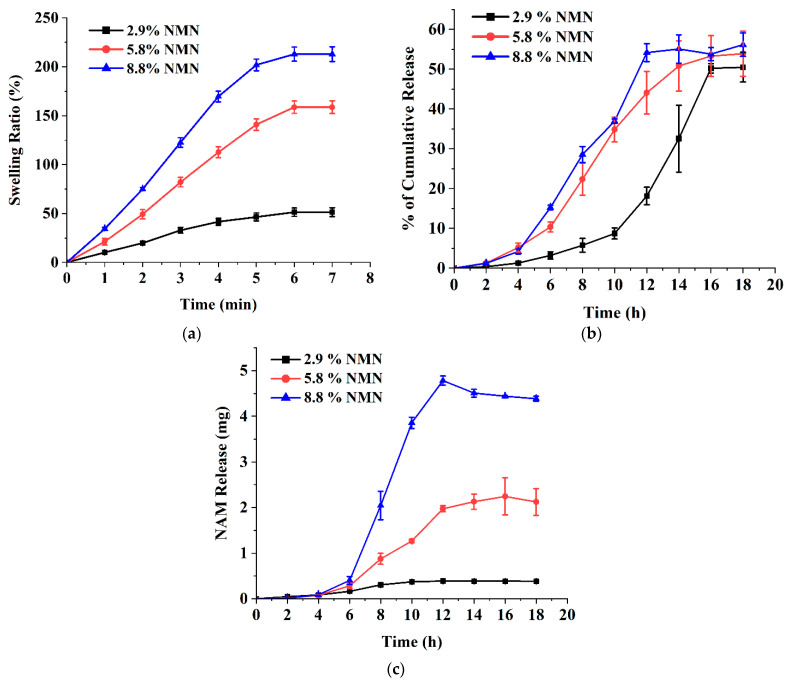
Effect of NMN concentration (2.9%, 5.8%, and 8.8% of PVA) on (**a**) swelling ratio (%), (**b**) NMN release (%), and (**c**) NAM production. All experiments were performed in triplicate. All graphs are showing errors as mean +/− standard deviation.

Figure 5a shows the effect of drug concentration on swelling ratio (%). After immersion of the microneedles in the liquid medium, the weight increased significantly from 51.4% swelling ratio at 2.9% NMN concentration in microneedles to 159% swelling ratio at 5.8% NMN concentration and to 213% at 8.8% NMN concentration. Similar results were reported for drug-loaded calcium pectinate gel beads [44]. As a result, it was found that the higher the drug content, the higher the swelling ratio. Drug release from matrices can be controlled by optimizing drug solubility, primary drug concentration in the matrix, polymer system, matrix porosity and flexibility, and matrix size and shape [45].

Figure 5b shows the effect of the NMN concentration on drug release from the microneedle patch. Increased NMN concentration in the microneedles resulted in slightly faster drug release. The cumulative release percentages of microneedles containing 2.9%, 5.8%, and 8.8% NMN were 50.48 ± 3.73%, 53.90 ± 5.67%, and 56.15 ± 2.94%, respectively. This result is also consistent with the results of other studies that evaluated the drug release of calcium pectinate gels prepared with different drug concentrations [46]. Increased drug concentration in the immersion solution and immersion time resulted in higher drug levels and faster drug release. The drug release data from HPLC showed another peak at a retention time of 3.5–4 min [47]. The new peak was found to be NAM based on a comparison of HPLC data of standard NAM, indicating that NAM is produced during NMN passes through the skin. Figure 5c shows the amount of produced NAM from NMN in transdermal release. NAM production may be the result of skin enzymatic hydrolysis. Porcine skin contains some hydrolysis enzymes such as uridine 5′-diphospho-glucuronosyltransferase, esterase/amidase, sulfotransferase, N-acetyltransferase, and glutathione S-transferase [48]. Figure 5b,c show that increasing the drug concentration in the patch also increases the NAM production.

In general, the hydrolysis rate of NMN was not high at low NMN loading, and the highest NAM production was 4.38 ± 0.05 mg in 8.8% of NMN microneedles. Therefore, it was decided to use a lower NMN concentration (2.9%) in the next step because there is less enzymatic hydrolysis during transdermal delivery.

**Figure 6:**
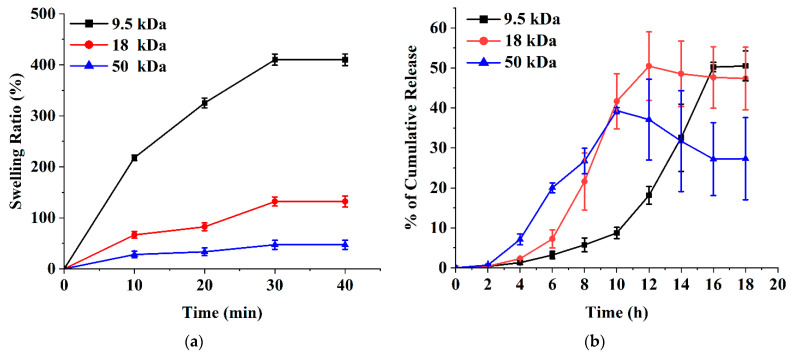
Effect of PVA molecular weights (9.5, 18, and 50 kDa) on (**a**) swelling ratio (%) and (**b**) NMN release (%). NMN concentration: 2.9% of PVA. All experiments were performed in triplicate. All graphs are showing errors as mean +/− standard deviation.

PVA is a synthetic biocompatible polymer approved by the US Food and Drug Administration, and its molecular weight can be systematically controlled. The properties of PVA depend on many factors, one of which is the molecular weight of the polymer. PVA gelation occurs due to the formation of PVA crystallites that act as physical crosslinks in the polymer network [35]. Polymer mixing, drug distribution, crystallinity, polymer molecular weight, and other factors are important to control the release profile [49,50]. The microneedles were made of PVA of different molecular weights. Figure 6a shows the effect of the PVA molecular weight (MW) on the swelling ratio. The microneedles performed a significant increase from a 47.2% swelling ratio at 50 kDa MW to a 410% swelling ratio at 9.5 kDa MW. The reason for the change in the swelling ratio at different molecular weights is that high molecular weight films can improve the physical crosslinking of polymer chains [51,52]. Another study [53] considered the effect of increasing the molecular weight of poly(ethylene oxide) (200–600 kDa) on the swelling ratio. The swelling ratio dropped from 1013% at 200 kDa MW to 800% at 400 kDa MW, and then slightly decreased to 780% at 600 kDa MW.

PVA-based microneedles could potentially have controlled release and could also be easily tuned by changing the molecular weight [54]. In general, high molecular weight PVA is expected to have a slower release rate than low molecular weight PVA due to its larger size and increased viscosity, which can hinder the diffusion of molecules through the polymer matrix [53]. However, the actual release rate may also depend on other factors such as the specific formulation, processing conditions, and the nature of the drug or active ingredient being released [54]. In some cases, high molecular weight PVA may actually have a faster release rate than the low molecular weight PVA. For example, if a high molecular weight PVA is more water soluble than a low molecular weight PVA, it will dissolve faster in the release medium and release the encapsulated molecules more rapidly [55]. Figure 6b shows the release profile of NMN at various molecular weights of PVA-based microneedles. PVA-based microneedles with an average molecular weight of 9.5 kDa released 50.48 ± 3.73% NMN within 18 h, whereas PVA-based microneedles with the average molecular weights of 18 and 50 kDa released 47.33 ± 7.83% and 27.30 ± 10.25%, respectively. The initial NMN release rate increased with increasing PVA molecular weight. A diffusion barrier may also form around the drug, slowing or preventing the release of the drug into the surrounding medium. Drugs can also undergo chemical changes over time that reduce their solubility or release rate [56]. On the other hand, the properties of the drug delivery system, such as particle size, shape, and composition, can affect the release rate of the drug. For example, if the particles are too large or too dense, they may not release the drug effectively [57].

**Figure 7:**
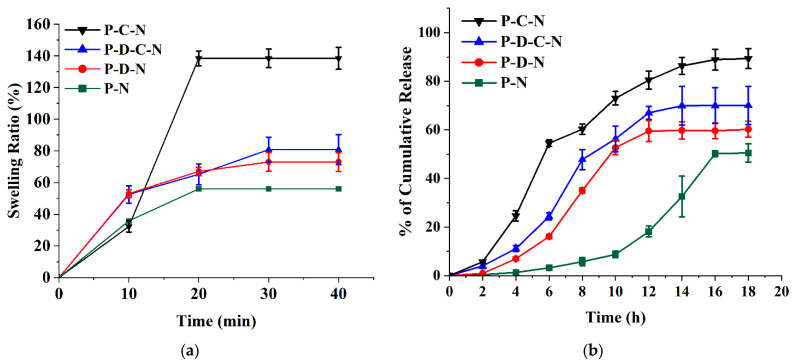
Effect of different compositions of PVA-based microneedles on (**a**) swelling ratio and (**b**) NMN release. NMN concentration: 2.9% of PVA, DMSO concentration: 128% of PVA, CMC concentration: 1.8% of PVA. P-C-N: PVA-CMC-NMN, P-D-C-N: PVA-DMSO-CMC-NMN, P-D-N: PVA-DMSO-NMN, P-N: PVA-NMN. All experiments were performed in triplicate. All graphs are showing errors as mean +/− standard deviation.

CMC is a water-soluble polymer and is a typical ionic cellulose ester with multiple carboxyl groups. PVA is a semi-crystalline polymer with hydroxyl groups that form intermolecular and intramolecular hydrogen bonds. A polymer blend can be defined as a mixture of two or more polymers or macromolecular compounds. The double-network polymer of PVA/CMC can be easily formed due to the excellent coordination and hydrophilicity of CMC [55]. Figure 7a,b shows the swelling ratio and release profiles of PVA patches of different compositions. PVA-NMN (P-N) and PVA-DMSO-NMN (P-D-N) swelled by 56% and 73%, respectively, and dissolved immediately after a certain period of time. The addition of DMSO increased the swelling ratio of the patch compared to the case without DMSO, but this composite also dissolved after some time due to the weak structure. The stability of the composite was increased by adding CMC to the polymer composition to form a double-network polymer structure. As can be seen in Figure 7a, PVA-CMC-NMN (P-C-N) showed a higher swelling ratio (138%). The presence of DMSO in the PVA structure causes the formation of a porous structure, which increases water penetration into the composite, resulting in higher swelling compared to the composite without DMSO [56].

Figure 7b shows the effect of CMC and DMSO along with PVA on NMN release. A relatively high release (89.34 ± 4.09% at 18 h) was obtained from a P-C-N matrix containing CMC as the blend polymer. The high drug dissolution/diffusion can be explained by the high-water solubility and hygroscopicity of CMC due to the presence of ionized carboxylic acid groups in the polymer structure. The latter can lead to an increase in the extent and rate of water uptake due to ion-pair repulsion, elongation of the gel network, and disruption of the bonds responsible for the gel structure [57]. The lowest NMN release was obtained in the P-N matrix (50.48 ± 3.73% at 18 h). This is due to the low resistance of PVA in alkaline solution and the absence of other polymers [47]. P-D-N and PVA-DMSO-CMC-NMN (P-D-C-N) both showed intermediate NMN release due to the inclusion of DMSO in the structure. DMSO improves bulk transport by reducing solution viscosity [58].

**Figure 8:**
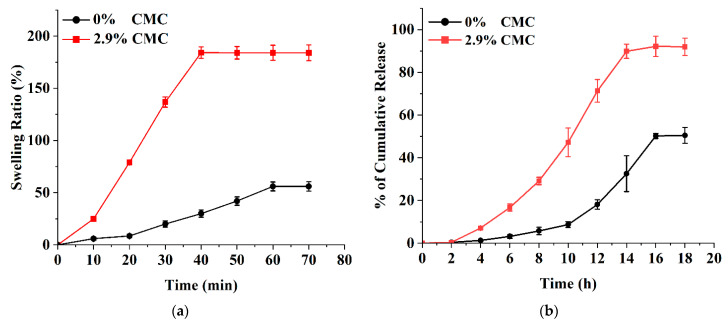
Effect of CMC concentrations (0% and 2.9% of PVA) on (**a**) swelling ratio and (**b**) NMN release. NMN concentration: 2.9% of PVA. All experiments were performed in triplicate. All graphs are showing errors as mean +/− standard deviation.

The swelling of the CMC-loaded microneedles is affected by the hydrophilicity of the carboxyl group in the polymer structure. Figure 8a shows the swelling pattern according to the use of 2.9% CMC in the PVA-based microneedle structure. There is a clear difference between composites with CMC and those without CMC. The results show that 2.9% CMC has a higher swelling ratio (186%) in comparison with the 0% CMC composite (48%). When a polymer containing CMC is immersed in an aqueous medium, the hydrophilic polymer chains create an osmotic pressure inside the hydrogel, causing the hydrogel matrix to swell. These results were consistent with other studies on the swelling of CMC [59]. CMC hydrogels were prepared using a crosslinking agent such as calcium chloride. The swelling study of CMC hydrogel showed that the swelling volume of the hydrogel was reduced by increasing the CMC content in the hydrogel [60]. Figure 8b shows the significant effect of CMC concentration on NMN release. Subsequent addition of CMC to the PVA matrix increased the NMN release and dissolution efficiency. The CMC-free PVA matrix showed lower release (50.48 ± 3.73% at 18 h). In contrast, a higher NMN release of 91.94 ± 4.03% was observed at 18 h with 2.9% CMC microneedles.

## 4. Conclusions

In this study, low molecular weight PVA (9.5, 18, 50 kDa) was used to form uniform sharp microneedles. The swelling and NMN release of the microneedle could be controlled by changing the NMN concentration, PVA molecular weight, and composition of the microneedle. PVA-based microneedles blended with CMC and DMSO showed higher NMN release compared to pure PVA microneedles. Higher drug concentration in the microneedle resulted in slightly increased NMN release, but hydrolysis of NMN to NAM was observed at higher drug concentration, suggesting that the drug concentration should be determined to lower drug hydrolysis. All formulations of microneedles exhibited the ability to release NMN through porcine skin, and the modification resulted in higher drug release. This study shows that PVA-based microneedles can be used in transdermal delivery of NMN as a patient-friendly delivery method.

## Data Availability

Data will be available on request.
